# Long non-coding RNA CDKN2B-AS1 regulates high glucose-induced human mesangial cell injury via regulating the miR-15b-5p/WNT2B axis

**DOI:** 10.1186/s13098-020-00618-z

**Published:** 2020-12-09

**Authors:** Jing Chang, Yanming Yu, Zhan Fang, Haiyan He, Dan Wang, Jian Teng, Lina Yang

**Affiliations:** 1 Department of Nephrology, Yantaishan Hospital, Yantai, Shandong China; 2 Department of Nephrology, Yantai Yuhuangding Hospital, No. 20 Yuhuangding East Road, Yantai, 264000 Shandong China

**Keywords:** DN, CDKN2B-AS1, miR-15b-5p, WNT2B

## Abstract

**Background:**

Long non-coding RNA cyclin-dependent kinase inhibitor 2B antisense RNA 1 (CDKN2B-AS1) has been reported to be related to diabetic nephropathy (DN) progression. However, the regulatory mechanisms of CDKN2B-AS1 in DN are unclear.

**Methods:**

High glucose (HG) was used to induce human mesangial cells (HMCs) for establishing the DN model. Expression levels of CDKN2B-AS1, microRNA (miR)-15b-5p, wingless-Type family member 2B (WNT2B) mRNA in serum and HMCs were detected through quantitative real-time polymerase chain reaction (qRT-PCR). The viability and cell cycle progression of HMCs were determined with Cell Counting Kit-8 (CCK-8) or flow cytometry assays. The levels of several proteins and inflammatory factors in HMCs were analyzed by western blotting or enzyme-linked immunosorbent assay (ELISA). The relationship between CDKN2B-AS1 or WNT2B and miR-15b-5p was verified with dual-luciferase reporter assay.

**Results:**

CDKN2B-AS1 and WNT2B were upregulated while miR-15b-5p was downregulated in serum of DN patients and HG-treated HMCs. CDKN2B-AS1 inhibition reduced HG-induced viability, cell cycle progression, ECM accumulation, and inflammation response in HMCs. CDKN2B-AS1 regulated WNT2B expression via competitively binding to miR-15b-5p. MiR-15b-5p inhibitor reversed CDKN2B-AS1 knockdown-mediated influence on viability, cell cycle progression, ECM accumulation, and inflammation response of HG-treated HMCs. The repressive effect of miR-15b-5p mimic on viability, cell cycle progression, ECM accumulation, and inflammation response of HG-treated HMCs was abolished by WNT2B overexpression.

**Conclusion:**

CDKN2B-AS1 regulated HG-induced HMC viability, cell cycle progression, ECM accumulation, and inflammation response via regulating the miR-15b-5p/WNT2B axis, provided a new mechanism for understanding the development of DN.

## Introduction

Diabetic nephropathy (DN) is a common cause of end-stage renal disease [1]. DN is characterized by mesangial hypertrophy, which is caused by mesangial cell proliferation and excessive accumulation of extracellular matrix (ECM) [2, 3]. Also, about 20–50% of people with diabetes will develop DN [4]. At present, conventional DN treatment measures include controlling blood sugar and blood pressure, and using inhibitors of the renin–angiotensin–aldosterone system, but this only slows the progression of DN without stopping or reversing it [5]. Studies have revealed that high glucose (HG) can induce mesangial cell ECM accumulation, proliferation, and inflammation response [6–8]. Hence, exploring the mechanisms that regulate the process of mesangial cells in DN is of great significance for the development of DN therapeutic strategies.

Long non-coding RNAs (lncRNAs) are a vital type of transcripts (over 200 nucleotides) that act as key players in development, health, and disease [9]. Increasing studies have proved that lncRNAs play important roles in kidney disease [10]. For instance, lncRNA TSI was revealed to impede renal fibrogenesis through modulating the TGF-β/Smad3 pathway [11]. Also, lncRNA HOTAIR could accelerate urine-derived sepsis-induced kidney injury [12]. Long non-coding RNA cyclin-dependent kinase inhibitor 2B antisense RNA 1 (CDKN2B-AS1) has been reported to be implicated in tumor advancement [13], atherosclerosis [14], cerebral infarction [15], diabetes [16], and so on. It has been revealed that CDKN2B-AS1 contributes to the progression of DN [16, 17]. However, the regulatory mechanisms of CDKN2B-AS1 in DN have not been completely clarified.

MicroRNAs (miRs) are short non-coding RNAs that regulate physiological and pathological processes through modulating the expression of target genes [18]. Mounting researches have revealed that miRs dysregulation is associated with diverse disease states, including kidney disease [19]. For example, the miR-17 family facilitated the advancement of polycystic kidney disease through the regulation of mitochondrial metabolism [20]. MiR-34a could contribute to renal fibrosis through decreasing klotho expression in HK-2 cells [21]. It was reported that miR-15b-5p exerted a protective role in DN [22, 23]. As far as we know, the targeting relationship between CDKN2B-AS1 and miR-15b-5p in DN is unclear.

Wingless-Type family member 2B (WNT2B), also termed as WNT13, exert a crucial role in Wnt/β-catenin pathway, which regulates modulate cell-to-cell interactions in physiological and pathological conditions, such as fibrosis, angiogenesis, and inflammation [24, 25]. WNT2B was revealed to facilitate HG-induced ECM and inflammation in MMCs [26]. However, the molecular mechanism of WNT2B-associated DN progression is unclear.

In the current study, we discovered that CDKN2B-AS1 silencing decreased viability, ECM accumulation, inflammation response, and cell cycle progression of human mesangial cells (HMCs) induced by HG through modulating the miR-15b-5p/WNT2B axis.

## Materials and methods

### Serum samples

The blood samples of 34 healthy controls (normal) and 34 DN patients were collected from the Yantaishan Hospital. The serum samples were obtained by centrifugation under 2000×*g* condition. The recruited healthy controls did not suffer from DN, autoimmune diseases, inflammatory diseases, or diabetes. The research was approved by the Ethics Committee of Yantaishan Hospital. Each participant signed a written informed consent before collecting blood samples. The clinical characteristics of DN patients and healthy controls were displayed in Table 1.

**Table 1:** Clinical characteristics of DN patients and healthy controls

Parameters	Normal group (n = 34)	DN group (n = 34)
Gender (male/female)	18/16	20/14
Age (years)	53.5 ± 4.3	56.2 ± 5.2
Duration of diabetes (years)		8.3 ± 2.1
Fasting plasma glucose (mmol/L)	4.5 ± 0.8	7.9 ± 2.1
Blood urea nitrogen (mmol/L)	4.2 ± 0.9	8.8 ± 3.7
Total cholesterol (mmol/L)	1.61 ± 0.6	2.37 ± 1.2
BMI	23.2 ± 0.7	30.6 ± 1.4
Creatinine (µmol/L)	81.6 ± 2.8	88.5 ± 6.0

### Cell culture and treatment

HMCs were purchased from Mingzhou Biological Technology Co., Let. (Ningbo, China). The cells were cultured in Dulbecco’s modified Eagle’s medium (DMEM) (Sigma, St Louis, MO, USA) containing glucose (5 mM, Sigma), fetal bovine serum (FBS, 10%, Solarbio, Beijing, China), and streptomycin/penicillin (1%, Solarbio) in a moist atmosphere with 5% CO_2_ at 37 °C. HMCs were cultured in DMEM containing HG (30 mM) for 24 h to simulate the DN status in vitro, and DMEM containing glucose (5 mM) was used as a control.

### Cell transfection

For cell transfection, the Lipofectamine 3000 reagent (Invitrogen, Carlsbad, CA, USA) was applied to transfect the vectors or oligonucleotides into HMCs (grown to 60–70%). Small interfering RNA (si) targeting CDKN2B-AS1 (si-CDKN2B-AS1) and corresponding negative control (si-NC) were synthesized by GenePharma (Shanghai, China). For pcDNA3.1-CDKN2B-AS1 (CDKN2B-AS1) or pcDNA3.1-WNT2B (WNT2B) generation, the full-length sequence of CDKN2B-AS1 or WNT2B was cloned into the pcDNA3.1 vectors (pcDNA) (Life Technologies, Grand Island, NY, USA). MiR-15b-5p mimic and inhibitor (miR-15b-5p and anti-miR-15b-5p) and their matched negative controls (miR-NC and anti-miR-NC) were purchased from RiboBio (Guangzhou, China).

### Quantitative real-time polymerase chain reaction (qRT-PCR)

Total RNA of serum samples and HMCs was extracted with the TRIzol reagent (Invitrogen). For complementary DNA generation, total RNA was reversely transcribed with Moloney Murine Leukemia Virus (M-MLV) First Strand Kit (Life Technologies) or miScript Reverse Transcription Kit (Qiagen, Hilden, Germany). The synthesized complementary DNA was utilized for qPCR with the SYBR Green (Promega, Madison, WI, USA) on Bio-Rad CFX96 Real-time PCR Systems (Bio-Rad, Hercules, CA, USA). Relative expression levels were figured with the 2^−ΔΔCt^ method, and glyceraldehyde-3-phosphate dehydrogenase (GAPDH), β-actin, or U6 small nuclear RNA (U6) was used as an internal control for CDKN2B-AS1, WNT2B, and miR-15b-5p. The primers were used as follows: GAPDH (F:5′-GACTCCACTCACGGCAAATTCA-3′; R:5′-TCGCTCCTGGAAGATGGTGAT-3′), CDKN2B-AS1 (F:5′-ACAGAAGCCTACGAAGAACTC-3′; R:5′-TGCATGGTGGTGCATCTGTA-3′), WNT2B (F:5′-GGGGCACGAGTGATCTGTG-3′; R:5′-GCATGATGTCTGGGTAACGCT-3′), U6 (F:5′-GCTCGCTTCGGCAGCACA-3′; R:5′-GAGGTATTCGCACCAGAGGA-3′), miR-15b-5p (F:5′-ATCCAGTGCGTGTCGTG-3′; R:5′-TGCTTAGCAGCACATCATG-3′), and β-actin (F:5′-AAATCTGGCACCACACCTTC-3′; R:5′-GGGGTGTTGAAGGTCTCAAA-3′).

### Cell viability analysis

HMCs (5 × 10^3^ cells/well) were seeded to a 96-well plate. After culture for 48 h, CCK-8 solution (10 μL, Solarbio) was added to each well and incubated for 2 h. Subsequently, the Microplate Reader (Bio-Rad) was used to measure the absorbance at 450 nm.

### Flow cytometry assay

For cell cycle assessment, HMCs were harvested and fixed with ethanol (70%). Subsequently, the cells were incubated with propidium iodide (PI) (50 μg/mL, Solarbio) and RNase A (100 μg/mL, Solarbio). Next, cell distribution was analyzed using a FACScan flow cytometry (BD Biosciences, Franklin lakes, NY, USA) with FACS Diva Software (BD Biosciences).

### Western blotting

Total protein of HMCs was extracted with the RIPA lysis buffer (Solarbio). Total protein was isolated by using sodium dodecyl sulfate–polyacrylamide gel electrophoresis (8–10%, SDS-PAGE). Thereafter, the separated proteins were electrophoretically transferred to the polyvinylidene fluoride (PVDF, Bio-Rad) membrane. Next, the membranes were blocked with Tris Buffered Saline Tween (TBST) buffer containing 5% non-fat milk. Then, the membranes were incubated with primary antibodies, including anti-proliferating cell nuclear antigen (PCNA) (sc-25280), anti-CyclinD1 (sc-450), anti-WNT2B (sc-166502), anti-Fibronectin (sc-18825), anti-Collagen IV (sc-59814), anti-GAPDH (sc-365062), and anti-β-actin (sc-47778). Next, the PVDF membrane was incubated with mouse-IgGκ BP-HRP (sc-516102). All antibodies were purchased from Santa Cruz Biotechnology (Santa Cruz, CA, USA). GAPDH and β-actin were used as internal references. The immunoblot was visualized through enhanced chemiluminescence solution (Solarbio).

### Enzyme-linked immunosorbent assay (ELISA)

After culture for 48 h, the supernatants of HMCs were collected. The levels of interleukin-6 (IL-6), interleukin-1β (IL-1β), and tumor necrosis factor-alpha (TNF-α) in supernatants were assessed with the ELISA kits (R&D Systems, Minneapolis, MN, USA). The concentrations of IL-6, IL-1β, and TNF-α were assessed with the Microplate Reader (Bio-Rad).

### Dual-luciferase reported assay

The binding sites of CDKN2B-AS1 or WNT2B in miR-15b-5p were predicted with the starBase database. For luciferase reporter plasmid generation, the fragments of wild type CDKN2B-AS1 (WT-CDKN2B-AS1), mutant CDKN2B-AS1 (MUT-CDKN2B-AS1), wild type 3′ untranslated regions (UTR) of WNT2B (WNT2B 3′UTR-WT), or mutant 3′UTR of WNT2B (WNT2B 3′UTR-MUT) containing miR-15b-5p binding sites were synthesized and inserted into the psiCHECK-2 vectors (Promega), respectively. Next, HMCs were cotransfected with the luciferase reporter plasmids and miR-NC or miR-15b-5p. After transfection for 48 h, the luciferase reporter assay system (Promega) was applied to assess the luciferase intensities for firefly and Renilla. The relative luciferase intensity was assessed by normalizing the firefly luminescence to Renilla luminescence.

### Statistical analysis

All experiments were repeated three times, and each experiment was performed in triplicate. Data exhibited as the mean ± standard deviation. Statistical analysis was implemented with SPSS 20.0 software (SPSS, Chicago, IL, USA). Differences were deemed significant if *P* < 0.05. The difference between 2 groups was evaluated with an unpaired Student’s *t* test. The differences among 3 or more groups were analyzed by one-way variance analysis (ANOVA) with Turkey’s post hoc test. The correlation between miR-15b-5p and CDKN2B-AS1 or WNT2B was assessed by Pearson’s correlation analysis.

## Results

### CDKN2B-AS1 was upregulated in DN and HG-induced HMCs

**Fig. 1:**
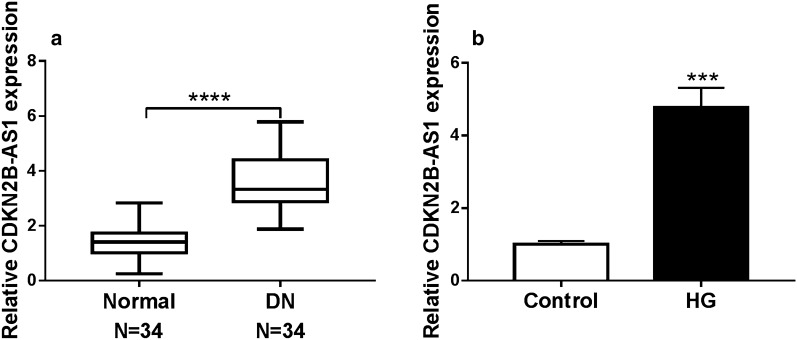
Expression levels of CDKN2B-AS1 in DN and HG-induced HMCs. **a** QRT-PCR was conducted to examine CDKN2B-AS1 expression in serum of 34 DN patients and 34 healthy controls, and GADPH was selected as an internal reference. **b** The expression levels of CDKN2B-AS1 in HMCs after HG (30 mM) or low glucose (5 mM) treatment were measured with qRT-PCR, and GADPH was selected as an internal reference. The experiments were repeated 3 times, and data were presented as mean ± standard deviation. ***P < 0.001 and ****P < 0.0001

To investigate the biological role of CDKN2B-AS1 in DN, we examined the levels of CDKN2B-AS1 in serum of 34 DN patients and 34 healthy controls with qRT-PCR. In contrast to the healthy controls, CDKN2B-AS1 expression was elevated in the serum of DN patients (Fig. 1a). Next, we detected the levels of CDKN2B-AS1 in HMCs after HG treatment. The results exhibited that CDKN2B-AS1 expression was increased in HMCs after HG treatment relative to the control group (Fig. 1b). These indicated that high CDKN2B-AS1 expression might be associated with DN development.

### Inhibition of CDKN2B-AS1 reduced cell viability, ECM accumulation, and inflammation response, and induced cell cycle arrest in HMCs under HG treatment

**Fig. 2:**
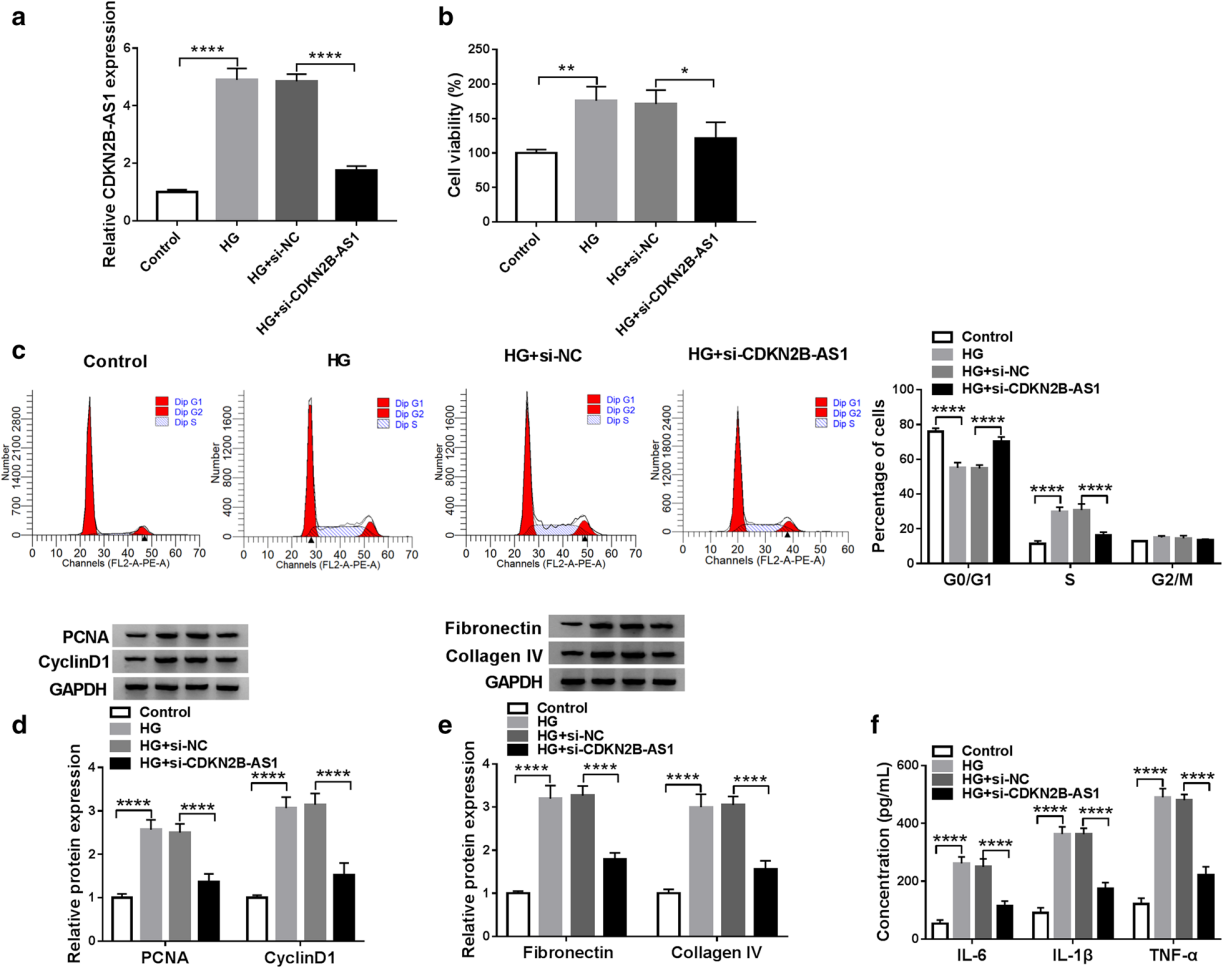
Effects of CDKN2B-AS1 inhibition on the viability, cell cycle progression, ECM accumulation, and inflammation response of HG-treated HMCs. **a**–**f** HMCs were transfected with si-NC or si-CDKN2B-AS1. **a** The expression CDKN2B-AS1 in HMCs under HG treatment was assessed with qRT-PCR, and GADPH was selected as an internal reference. **b**, **c** The viability and cell cycle progression of HMCs under HG treatment were evaluated by CCK-8 or flow cytometry assays. **d**, **e** After HG treatment, the levels of PCNA, CyclinD1, Fibronectin, and Collagen IV in HMCs were detected by western blotting, and GADPH was selected as an internal control. **f** The levels of IL-6, IL-1β, and TNF-α in HMCs after HG treatment were measured by ELISA. The experiments were repeated 3 times, and data were presented as mean ± standard deviation. *P < 0.05, **P < 0.01, and ****P < 0.0001

Next, we performed loss-of-function experiments to explore the role of CDKN2B-AS1 in DN. QRT-PCR displayed that the elevation of CDKN2B-AS1 in HMCs under HG treatment was reversed after si-CDKN2B-AS1 transfection compared to the control si-NC (Fig. 2a). In the next step, we explored the influence of CDKN2B-AS1 inhibition on the viability, cell cycle progression, ECM accumulation, inflammation response of HMCs under HG treatment. CCK-8 assay exhibited that the viability of HMCs was increased under HG treatment, but this tendency was restored by CDKN2B-AS1 inhibition (Fig. 2b). Flow cytometry assay showed that HG treatment reduced cell number in G0/G1 stage and elevated cell number in S stage in HMCs, while this influence was abolished by CDKN2B-AS1 silencing (Fig. 2c). Western blotting exhibited that silenced CDKN2B-AS1 expression reversed the upregulation of PCNA, CyclinD1, Fibronectin, and Collagen IV in HG-induced HMCs (Fig. 2d and e). Then, we detected the levels of pro-inflammatory markers including IL-6, IL-1β, and TNF-α in HMCs after HG treatment. Results of ELISA showed that HG treatment elevated the levels of IL-6, IL-1β, and TNF-α in HMCs, while this increase was abolished by CDKN2B-AS1 knockdown (Fig. 2f). These data indicated that CDKN2B-AS1 inhibition could reduce cell viability, ECM accumulation, and inflammation response, and induced cell cycle arrest in HG-treated HMCs.

### CDKN2B-AS1 acted as a sponge for miR-15b-5p in HMCs

**Fig. 3:**
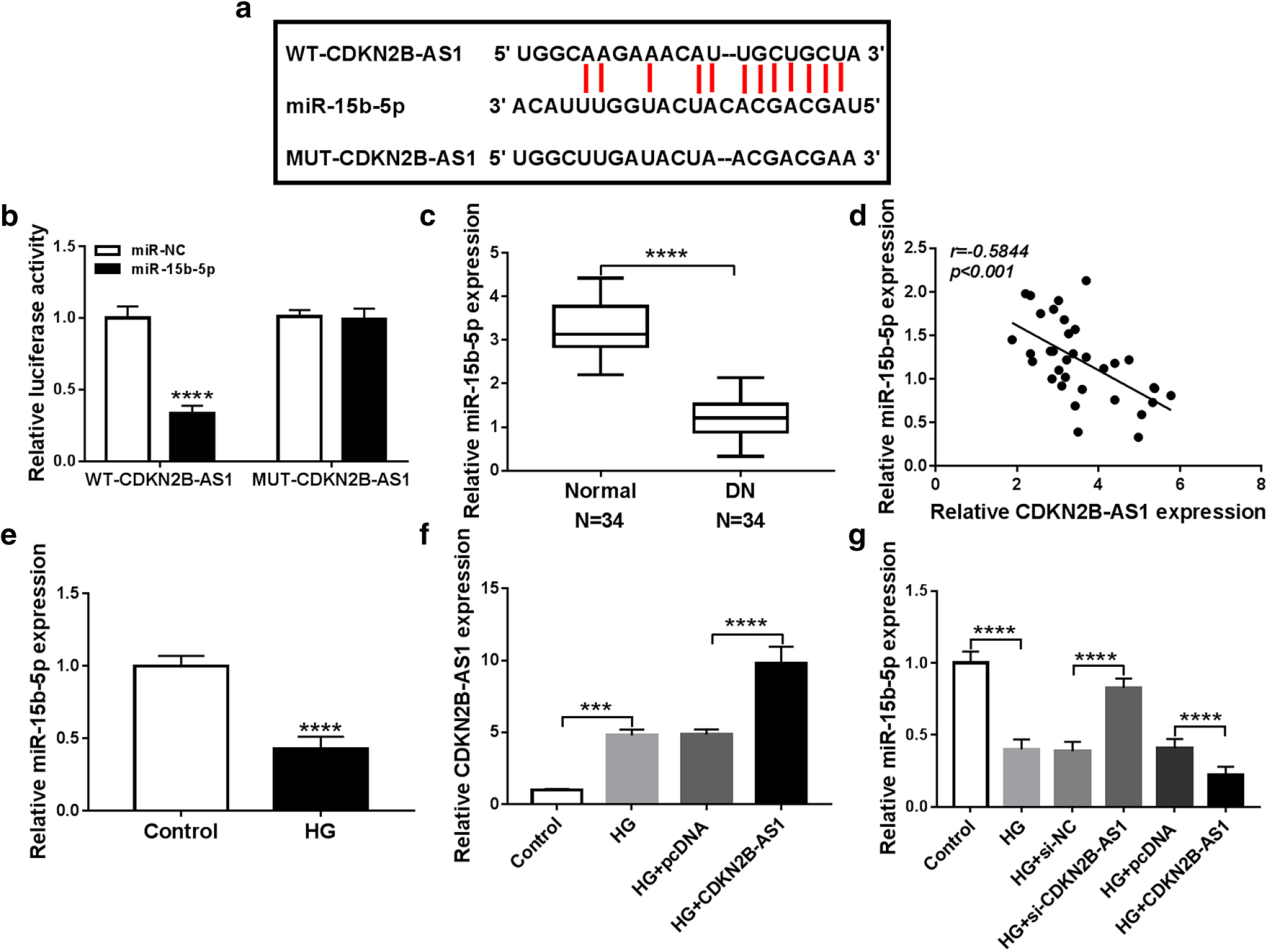
CDKN2B-AS1 was verified as a sponge for miR-15b-5p in HMCs. **a** Complementary binding sites within CDKN2B-AS1 and miR-15b-5p were predicted using the starBase database. **b** Dual-luciferase reporter assay was executed in HMCs cotransfected with miR-15b-5p or miR-NC and luciferase vectors containing WT-CDKN2B-AS1 or MUT-CDKN2B-AS1. **c** QRT-PCR was employed to assess the levels of miR-15b-5p in serum of 34 DN patients and 34 healthy controls, and U6 was selected as an internal reference. **d** Pearson’s correlation analysis displayed the correlation between CDKN2B-AS1 and miR-15b-5p in serum of DN patients. **e** QRT-PCR exhibited the expression of miR-15b-5p in HMCs after HG treatment, and U6 was selected as an internal reference. **f** QRT-PCR presented the expression of CDKN2B-AS1 in HMCs transfected with pcDNA or CDKN2B-AS1 under HG treatment, and GADPH was selected as an internal control. **g** QRT-PCR revealed the influence of CDKN2B-AS1 knockdown or CDKN2B-AS1 overexpression on the expression of miR-15b-5p in HMCs under HG treatment, and U6 was selected as an internal reference. The experiments were repeated 3 times, and data were presented as mean ± standard deviation. ***P < 0.001 and ****P < 0.0001

To explore the latent regulatory mechanism of CDKN2B-AS1 in DN, we predicted miRs that had possible binding sites for CDKN2B-AS1 through using starBase database. Based on bioinformatics, we discovered that miR-15b-5p possessed complementary binding sites with CDKN2B-AS1 (Fig. 3a). Dual-luciferase reporter assay displayed that miR-15b-5p overexpression reduced the luciferase intensity of luciferase vectors with WT-CDKN2B-AS1 in HMCs, but there was no overt difference in luciferase vectors with MUT-CDKN2B-AS1 (Fig. 3b). QRT-PCR exhibited that miR-15b-5p expression was reduced in serum of DN patients in comparison to the healthy controls (Fig. 3c). Moreover, CDKN2B-AS1 and miR-15b-5p expression had a negative correlation in serum of DN patients (Fig. 3d). Also, miR-15b-5p expression was reduced in HMCs after HG treatment (Fig. 3e). Furthermore, CDKN2B-AS1 expression was further elevated in HMCs after CDKN2B-AS1 transfection under HG treatment compared to the control pcDNA (Fig. 3f). We also discovered that CDKN2B-AS1 inhibition reversed the downregulation of miR-15b-5p in HMCs after HG treatment, but CDKN2B-AS1 overexpression further reduced miR-15b-5p expression in HMCs after HG treatment (Fig. 3g). These data suggested that CDKN2B-AS1 served as a sponge of miR-15b-5p in HMCs.

### MiR-15b-5p inhibitor reversed CDKN2B-AS1 silencing-mediated impacts on the viability, cell cycle progression, ECM accumulation, and inflammation response of HG-treated HMCs

**Fig. 4:**
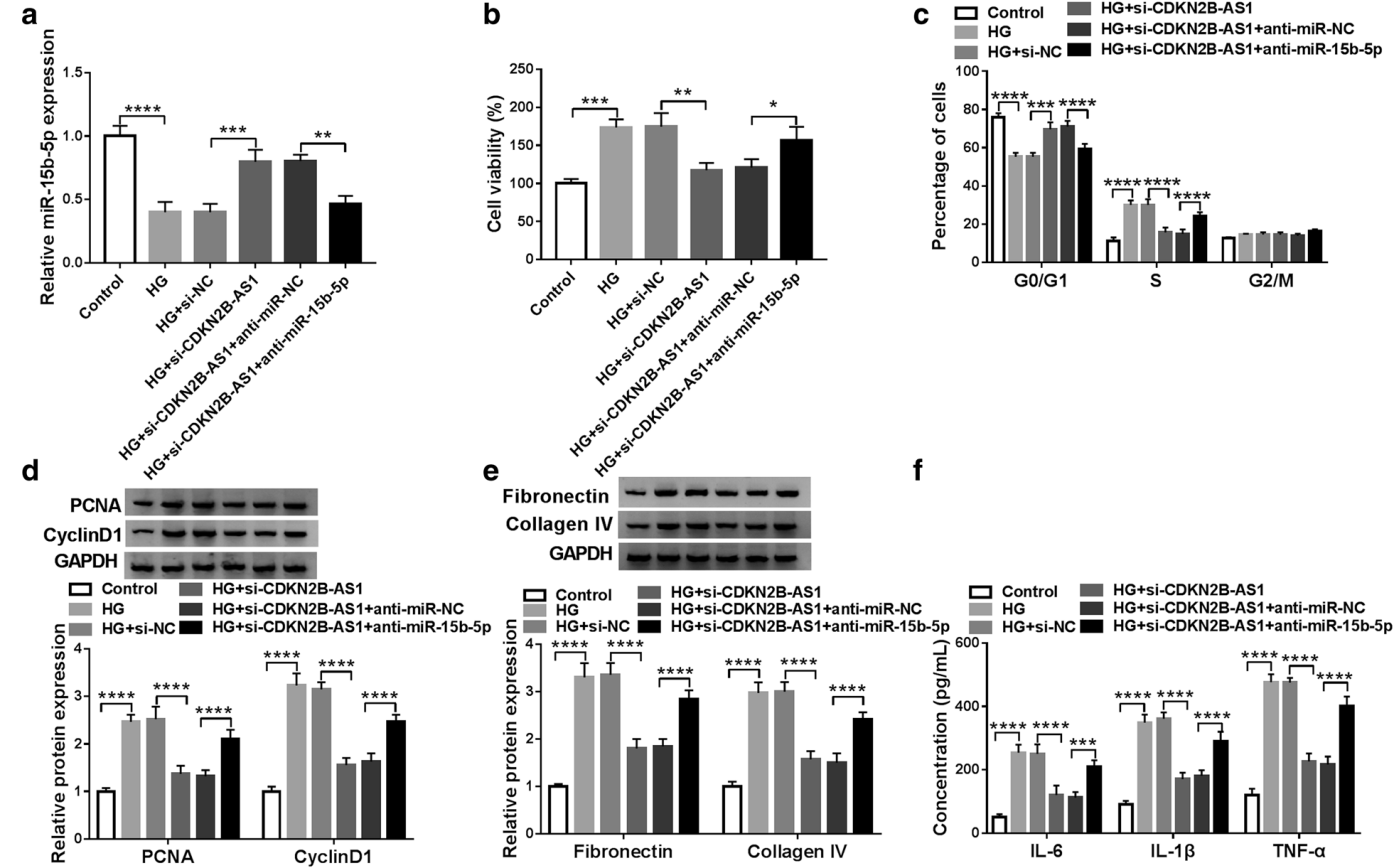
CDKN2B-AS1 regulated DN progression by sponging miR-15b-5p in HMCs. **a**–**f** HMCs were transfected with si-NC, si-CDKN2B-AS1, si-CDKN2B-AS1 + anti-miR-NC, or si-CDKN2B-AS1 + anti-miR-15b-5p. **a** Expression of miR-15b-5p in HG-treated HMCs was detected by qRT-PCR, and U6 was selected as an internal reference. **b**, **c** CCK-8 and flow cytometry assays were employed to analyze the proliferation or cell cycle progression of HG-treated HMCs. **d**, **e** Western blotting was executed to assess the levels of PCNA, CyclinD1, Fibronectin, and Collagen IV in HG-treated HMCs, and GADPH was selected as an internal control. **f** ELISA was conducted to evaluate the levels of IL-6, IL-1β, and TNF-α in HG-induced HMCs. The experiments were repeated 3 times, and data were presented as mean ± standard deviation. *P < 0.05, **P < 0.01, ***P < 0.001, and ****P < 0.0001

Based on the above findings, we further explored whether miR-15b-5p was related to the DN advancement mediated by CDKN2B-AS1. Results of qRT-PCR presented that the downregulation of miR-15b-5p in HG-treated HMCs was reversed by CDKN2B-AS1 knockdown, but this influence was partly abolished after anti-miR-15b-5p transfection (Fig. 4a). CCK-8 assay indicated that miR-15b-5p silencing reversed the decrease of viability of HMCs mediated by CDKN2B-AS1 silencing under HG treatment (Fig. 4b). Flow cytometry assay revealed that miR-15b-5p inhibitor overturned the arrest of cell cycle progression in HG-treated HMCs caused by CDKN2B-AS1 knockdown (Fig. 4c). Moreover, the downregulation of PCNA, CyclinD1, Fibronectin, and Collagen IV in HG-treated HMCs caused by CDKN2B-AS1 inhibition was abolished by miR-15b-5p knockdown (Fig. 4d and e). ELISA presented that silenced miR-15b-5p expression abrogated the reduction of IL-6, IL-1β, and TNF-α in HG-induced HMCs caused by CDKN2B-AS1 knockdown (Fig. 4f). These results suggested that CDKN2B-AS1 regulated DN development via sponging miR-15b-5p in HMCs.

### WNT2B acted as a target for miR-15b-5p in HMCs

**Fig. 5:**
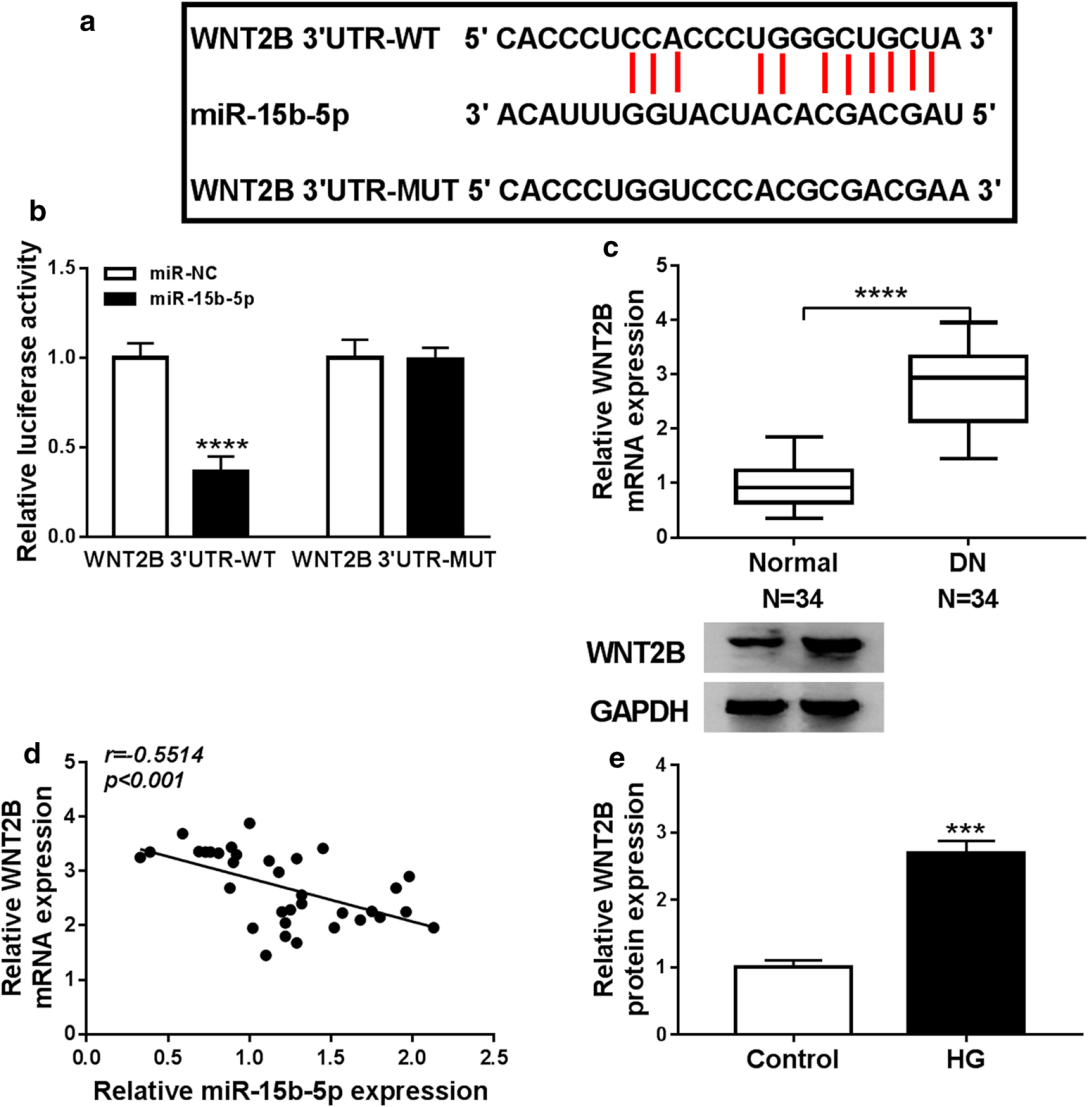
WNT2B was verified as a target for miR-15b-5p in HMCs. **a** Complementary binding sites between WNT2B and miR-15b-5p were predicted using the starBase database. **b** Dual-luciferase reporter assay revealed the luciferase activity in HMCs cotransfected with miR-15b-5p or miR-NC and luciferase vectors containing WNT2B 3′UTR-WT or WNT2B 3′UTR-MUT. **c** QRT-PCR exhibited the expression of WNT2B mRNA in serum of 34 DN patients and 34 healthy controls, and GADPH was selected as an internal control. **d** Pearson’s correlation analysis displayed the correlation between WNT2B mRNA and miR-15b-5p in serum of DN patients. **e** Western blotting exhibited the level of WNT2B protein in HG-induced HMCs, and GADPH was selected as an internal control. The experiments were repeated 3 times, and data were presented as mean ± standard deviation. ***P < 0.001 and ****P < 0.0001

In view of the above results, we further investigated the downstream target of miR-15b-5p in HMCs. Online bioinformatic (starBase) prediction exhibited that WNT2B might be a target for miR-15b-5p (Fig. 5a). We also observed that the luciferase intensity in HMCs cotransfected with miR-15b-5p and luciferase vectors containing WNT2B 3′UTR-WT was apparently reduced, while the luciferase intensity did not change in HMCs cotransfected with miR-15b-5p and luciferase vectors containing WNT2B 3′UTR-MUT (Fig. 5b). Moreover, WNT2B mRNA level was upregulated in serum of DN patients compared to the healthy controls (Fig. 5c). Pearson’s correlation analysis exhibited that WNT2B mRNA expression was negatively correlated with miR-15b-5p in serum of DN patients (Fig. 5d). Furthermore, the level of WNT2B protein was elevated in HG-induced HMCs (Fig. 5e). Together, these data manifested that WNT2B acted as a target for miR-15b-5p in HMCs.

### WNT2B overexpression overturned miR-15b-5p mimic-mediated effects on the viability, cell cycle progression, ECM accumulation, and inflammation response of HG-treated HMCs

**Fig. 6:**
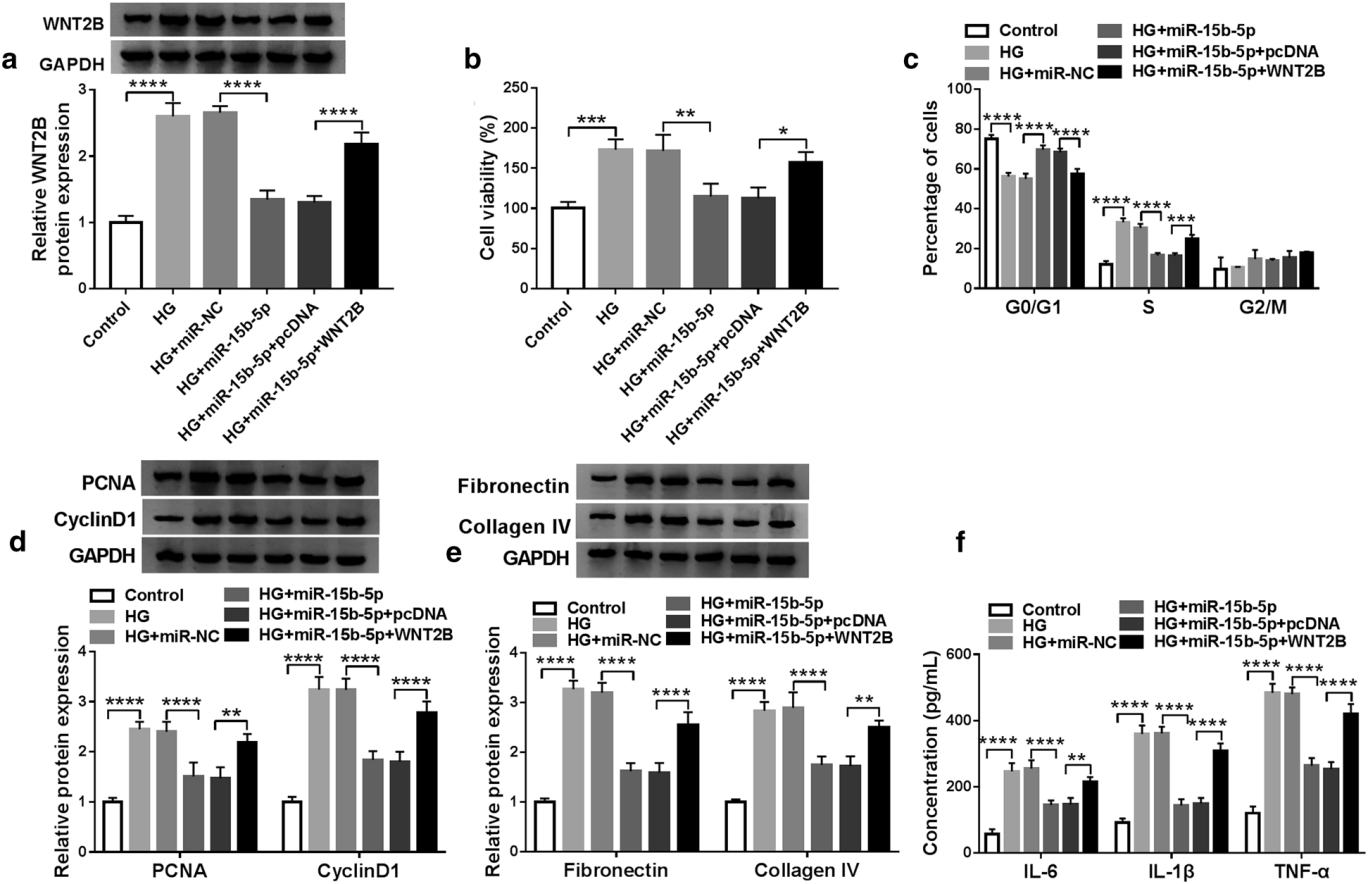
MiR-15b-5p regulated DN development through WNT2B. **a**–**f** HMCs were transfected with miR-NC, miR-15b-5p, miR-15b-5p + pcDNA, or miR-15b-5p + WNT2B. **a** The level of WNT2B protein in HMCs after HG treatment was detected with western blotting, and GADPH was selected as an internal control. **b**, **c** The viability and cell cycle progression of HMCs under HG treatment were determined via CCK-8 or flow cytometry assays. **d**, **e** The levels of PCNA, CyclinD1, Fibronectin, and Collagen IV in HMCs under HG treatment were evaluated by western blotting, and GADPH was selected as an internal control. **f** The levels of IL-6, IL-1β, and TNF-α in HMCs after HG treatment were analyzed by ELISA. The experiments were repeated 3 times, and data were presented as mean ± standard deviation. *P < 0.05, **P < 0.01, ***P < 0.001, and ****P < 0.0001

Given that miR-15b-5p targeted WNT2B in HMCs, we further investigated whether WNT2B was involved in the progression of DN regulated by miR-15b-5p. Western blotting presented that miR-15b-5p overexpression reversed the upregulation of WNT2B protein in HMCs after HG treatment, but this tendency was restored after WNT2B transfection (Fig. 6a). Also, miR-15b-5p elevation reduced the viability of HMCs caused by HG treatment, while this decrease was abolished by forcing WNT2B expression (Fig. 6b). Moreover, forced WNT2B expression reversed the repressive influence of miR-15b-5p mimic on cell cycle progression of HG-induced HMCs (Fig. 6c). Furthermore, WNT2B overexpression abolished the decrease of PCNA, CyclinD1, Fibronectin, and Collagen IV in HG-induced HMCs mediated by miR-15b-5p mimic (Fig. 6d and e). Additionally, overexpression of miR-15b-5p reduced the levels of IL-6, IL-1β, and TNF-α in HG-induced HMCs, while this impact was abolished by forcing WNT2B expression (Fig. 6f). Collectively, these data indicated that miR-15b-5p regulated DN development via targeting WNT2B.

### CDKN2B-AS1 regulated WNT2B expression via competitively binding to miR-15b-5p in HMCs

**Fig. 7:**
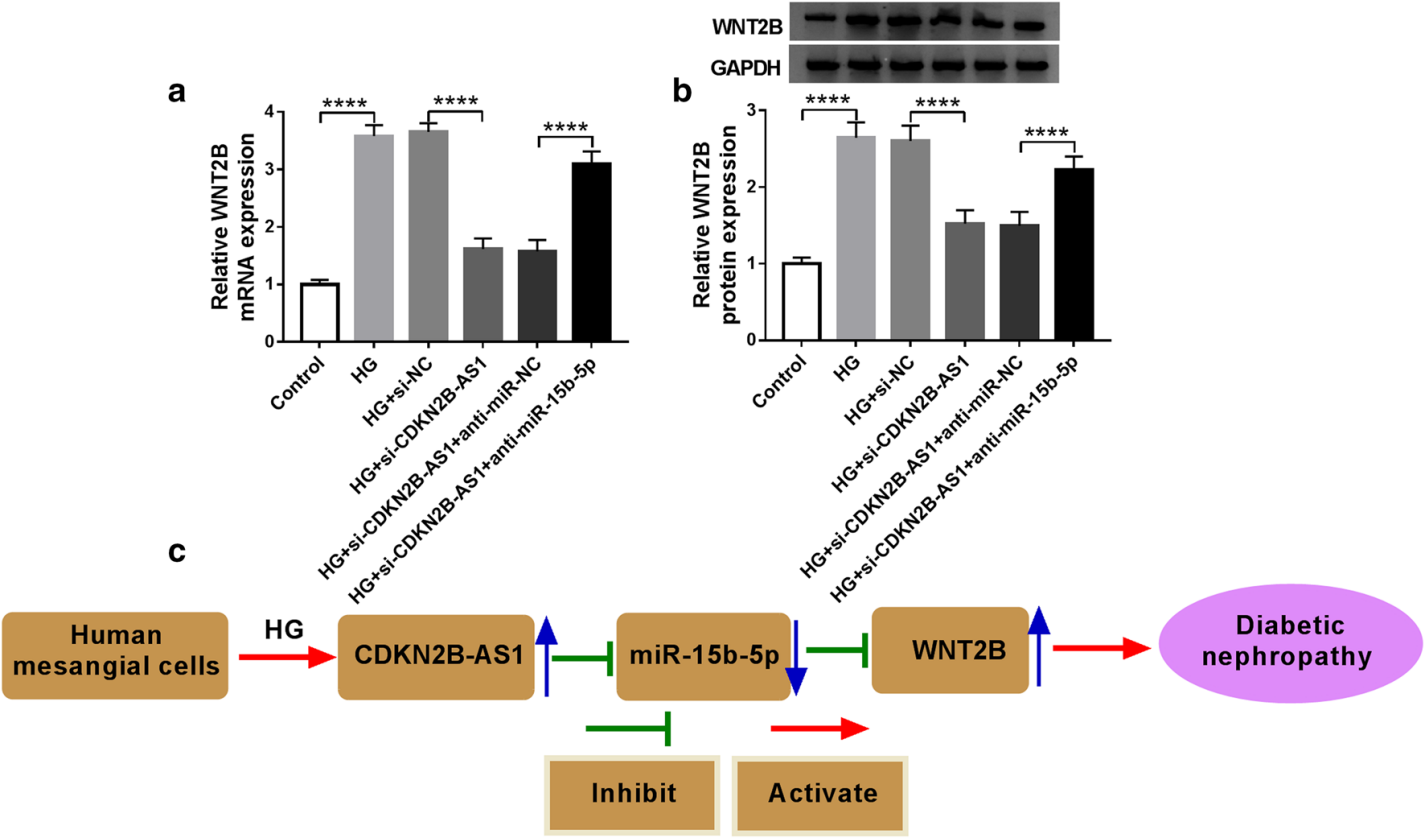
CDKN2B-AS1 regulated DN progression via modulating the miR-15b-5p/WNT2B axis. **a**, **b** After si-NC, si-CDKN2B-AS1, si-CDKN2B-AS1 + anti-miR-NC, or si-CDKN2B-AS1 + anti-miR-15b-5p transfection, the levels of WNT2B mRNA and protein in HMCs under HG treatment were detected by qRT-PCR or western blotting, and GADPH was selected as an internal reference. **c** Schematic diagram showing CDKN2B-AS1 regulated DN development through regulation of the miR-15b-5p/WNT2B axis. The experiments were repeated 3 times, and data were presented as mean ± standard deviation. ****P < 0.0001

Considering that CDKN2B-AS1 served as a sponge for miR-15b-5p, which targeted WNT2B in HMCs, we further explored whether CDKN2B-AS1 functioned as a ceRNA in DN progression. The results presented that CDKN2B-AS1 knockdown reduced the levels of WNT2B mRNA and protein in HG-induced HMCs, while this impact was reversed by miR-15b-5p inhibition (Fig. 7a and b). Considering that GAPDH plays an important role in diabetic lesions [27, 28], we used β-actin as an internal reference gene to further verify the levels of WNT2B mRNA and protein in HG-treated HMCs. As exhibited in Additional file 1: Fig. S1, the upregulation of WNT2B mRNA and protein in HG-treated HMCs was reversed after si-CDKN2B-AS1 transfection. However, this influence was antagonized by repressing expression of miR-15b-5p. The results were consistent with Fig. 7a and b, which results were obtained using GADPH as an internal reference, indicating that GADPH as an internal reference had little effect on the results of this study. Therefore, these data suggested that CDKN2B-AS1 regulated DN progression via modulating WNT2B expression via binding to miR-15b-5p (Fig. 7c).

## Discussion

Mounting studies have indicated that lncRNAs can act as diagnostic biomarkers and therapeutic targets in some diseases, including DN [29]. CDKN2B-AS1 exerted a vital role in the advancement of a range of diseases. Previous research pointed out that CDKN2B-AS1 could impede ADAM10 expression in atherosclerosis, which decreased inflammation response and contributed to cholesterol efflux [14]. In several tumors, reduced CDKN2B-AS1 expression could repress cancer cell proliferation and cell cycle proliferation [30, 31]. Another research indicated that CDKN2B-AS1 decreased the protective role of Rhein via elevating inflammation response in uric acid nephropathy rats [32]. Deng et al. suggested that CDKN2B-AS1 knockdown reduced the inflammation response of lipopolysaccharide-treated HK-2 cells via increasing miR-9 expression [33]. Herein, CDKN2B-AS1 silencing decreased viability, ECM accumulation, inflammation response, and induced cell cycle arrest of HG-induced HMCs. Li et al. revealed that CDKN2B-AS1 silencing curbed ECM accumulation and proliferation of HG-induced HMCs through regulating the miR-424-5p/HMGA2 pathway in DN [17]. Also, CDKN2B-AS1 interference played a protective effect on diabetic mouse kidneys [16]. These data indicated that CDKN2B-AS1 acted as an unfavorable factor in HG-induced HMCs injury.

Accumulated researches have proved that CDKN2B-AS1 takes part in the regulation of the gene expression via serving as a ceRNA [17, 30]. One report claimed that miR-15b-5p could reduce HG-induced podocyte injury by repressing inflammation response, oxidative stress, and apoptosis of podocyte via downregulating Sema3A [22]. Shen et al. revealed that miR-15b-5p overexpression could reduce apoptosis in human kidney cells induced by HG [23]. Herein, CDKN2B-AS1 was proved as a sponge for miR-15b-5p in HMCs. Moreover, miR-15b-5p inhibitor reversed the repressive impact of CDKN2B-AS1 knockdown on viability, ECM accumulation, inflammation response, and cell cycle progression of HG-induced HMCs. These data manifested that miR-15b-5p exerted a protective role in HG-induced HMCs injury, and CDKN2B-AS1 regulated HG-induced HMCs injury via sponging miR-15b-5p.

Additionally, we found that WNT2B acted as a downstream target of miR-15b-5p in HMCs. Forced WNT2B expression abolished the inhibitory influence of miR-15b-5p mimic on the viability, ECM accumulation, inflammation response, and cell cycle progression of HG-induced HMCs. It was reported that WNT2B was expressed in kidney ontogeny [34]. Recent research indicated that lncRNA Hottip knockdown reduced HG-induced ECM accumulation and inflammation response in MMCs through inhibiting WNT2B expression by sponging miR-455-3p [26]. These data indicated that WNT2B served as an unfavorable gene in DN. Also, CDKN2B-AS1 regulated WNT2B expression via sponging miR-15b-5p in HMCs. Therefore, we concluded that CDKN2B-AS1 regulated the progression of DN via the miR-15b-5p/WNT2B axis. Unfortunately, we did not verify the regulatory mechanism of CDKN2B-AS1 in mice model in vivo and did not explore the downstream pathway of the CDKN2B-AS1/miR-15b-5p/WNT2B axis, which can be explored in the further.

Overall, these findings revealed that CDKN2B-AS1 silencing decreased HG-induced HMC viability, ECM accumulation, inflammation response, and cell cycle progression via regulating the miR-15b-5p/WNT2B axis. The research provided a new mechanism for the comprehension of the development of DN.

## Supplementary information

**Additional file 1: Fig. S1 Relative mRNA and prtoein levels of WNT2B in HMCs under HG treatment.** (A and B) Relative levels of WNT2B mRNA and protein in HG-treated HMCs transfected with si-NC, si-CDKN2B-AS1, si-CDKN2B-AS1 + anti-miR-NC, or si-CDKN2B-AS1 + anti-miR-15b-5p were measured by qRT-PCR or western blotting, and β-actin was selected as an internal reference. ***P < 0.001 and ****P < 0.0001.

## Data Availability

All data generated or analyzed during this study are included in this article.

## Abbreviations

CDKN2B-AS1: Cyclin-dependent kinase inhibitor 2B antisense RNA 1
DN: Diabetic nephropathy
HG: High glucose
WNT2B: Wingless-Type family member 2B
ELISA: Enzyme-linked immunosorbent assay
