# Supplementary figures and images for: Long non-coding RNA CDKN2B-AS1 regulates high glucose-induced human mesangial cell injury via regulating the miR-15b-5p/WNT2B axis
Source: Diabetol Metab Syndr. 2020 Dec 9;12:109. doi: 10.1186/s13098-020-00618-z (PMC7724838; doi:10.1186/s13098-020-00618-z)

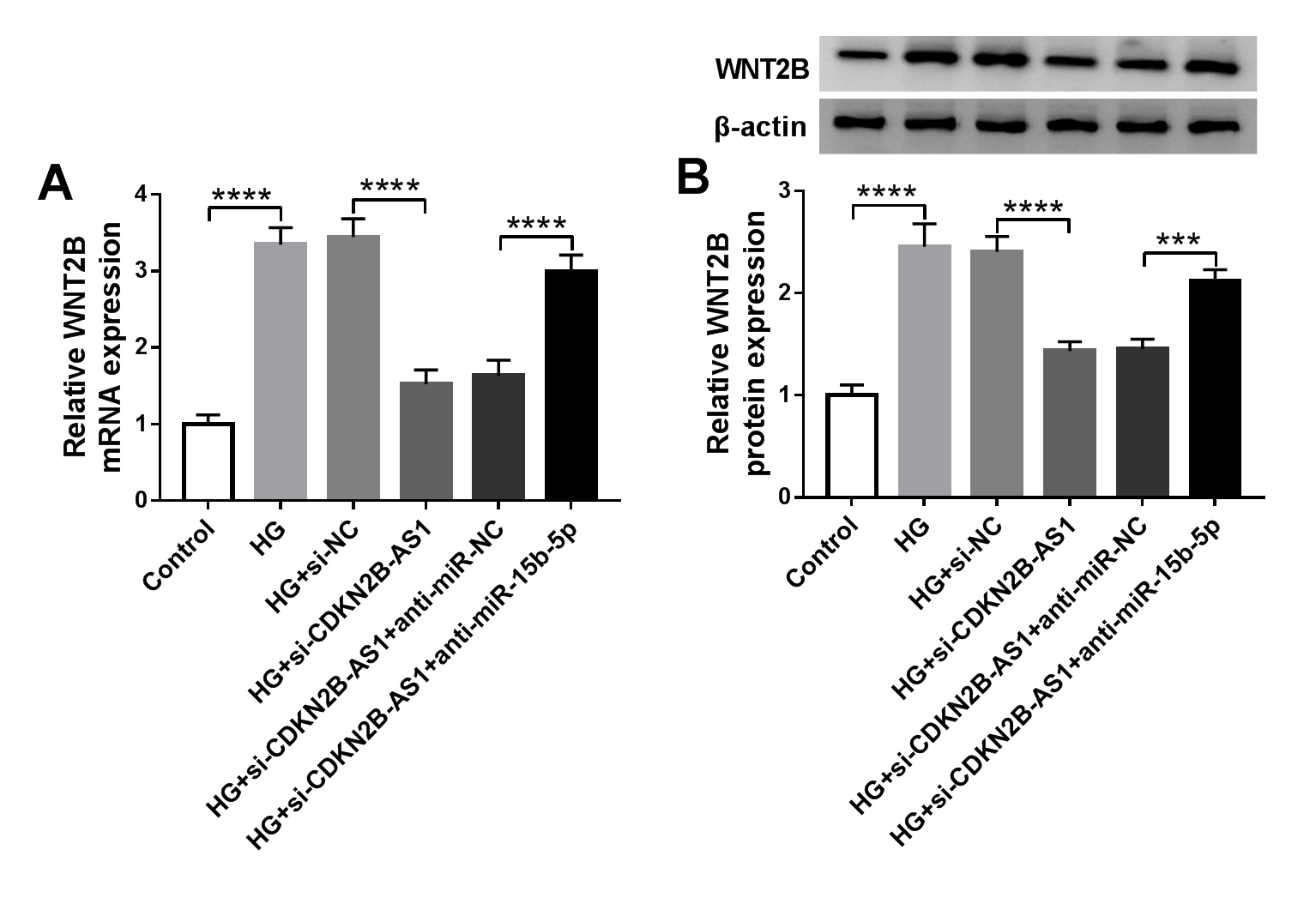

Supplement: Supplementary file 1 — Additional file 1: Fig. S1 Relative mRNA and prtoein levels of WNT2B in HMCs under HG treatment. (A and B) Relative levels of WNT2B mRNA and protein in HG-treated HMCs transfected with si-NC, si-CDKN2B-AS1, si-CDKN2B-AS1 + anti-miR-NC, or si-CDKN2B-AS1 + anti-miR-15b-5p were measured by qRT-PCR or western blotting, and β-actin was selected as an internal reference. ***P < 0.001 and ****P < 0.0001. [file 13098_2020_618_MOESM1_ESM.tif]
